# Achieving Surgical, Obstetric, Trauma, and Anesthesia (SOTA) care for all in South Asia

**DOI:** 10.3389/fpubh.2024.1325922

**Published:** 2024-02-21

**Authors:** Siddhesh Zadey, Shirish Rao, Isha Gondi, Natalie Sheneman, Chaitrali Patil, Anveshi Nayan, Himanshu Iyer, Arti Raj Kumar, Arun Prasad, G. Allen Finley, Chellapuram Raja Krishna Prasad, Chintamani, Dhananjaya Sharma, Dhruva Ghosh, Gnanaraj Jesudian, Irum Fatima, Jogi Pattisapu, Justin Sangwook Ko, Lovenish Bains, Mashal Shah, Mohammed Shadrul Alam, Narmada Hadigal, Naveen Malhotra, Nilmini Wijesuriya, Prateek Shukla, Sadaf Khan, Sunil Pandya, Tariq Khan, Tashi Tenzin, Venkat Raja Hadiga, Daniel Peterson

**Affiliations:** ^1^Association for Socially Applicable Research (ASAR), Pune, Maharashtra, India; ^2^Department of Epidemiology, Mailman School of Public Health, Columbia University, New York, NY, United States; ^3^GEMINI Research Center, Duke University School of Medicine, Durham, NC, United States; ^4^Dr. D.Y. Patil Medical College, Hospital, and Research Centre, Pune, Maharashtra, India; ^5^Global Alliance for Surgery, Obstetric, Trauma and Anaesthesia Care, Chicago, IL, United States; ^6^Seth G.S. Medical College and K.E.M. Hospital, Mumbai, Maharashtra, India; ^7^Department of Health and Human Sciences, Baylor University, Waco, TX, United States; ^8^Department of Biology and Statistics, George Washington University, Washington, DC, United States; ^9^India Hub, NIHR Health Research Unit On Global Surgery, Christian Medical College, Ludhiana, Punjab, India; ^10^Indraprastha Apollo Hospital, New Delhi, India; ^11^Department of Anesthesiology, Dalhousie University, Halifax, NS, Canada; ^12^Apollo Hospital, Hyderguda, Hyderabad, Telangana, India; ^13^Department of Surgery, Vardhman Mahavir Medical College Safdarjung Hospital, New Delhi, India; ^14^Department of Surgery, NSCB Government Medical College, Jabalpur, India; ^15^Karunya Rural Community Hospital Karunya Nagar, Coimbatore, Tamil Nadu, India; ^16^Association of Rural Surgeons of India, Wardha, India; ^17^International Federation of Rural Surgeons, Ujjain, India; ^18^Rural Surgery Innovations Private Limited, Dimapur, Nagaland, India; ^19^IRD Pakistan and the Global Surgery Foundation, Karachi, Sindh, Pakistan; ^20^University of Central Florida College of Medicine, Orlando, FL, United States; ^21^Samsung Medical Center, Sungkyunkwan University School of Medicine, Seoul, Republic of Korea; ^22^Department of Surgery, Maulana Azad Medical College and Lok Nayak Hospital, New Delhi, India; ^23^WHO Collaborating Centre for Research in Surgical Care Delivery in LMIC, Mumbai, Maharashtra, India; ^24^Department of Surgery, Aga Khan University, Karachi, Sindh, Pakistan; ^25^Department of Pediatric Surgery, Mugda Medical College, Dhaka, Bangladesh; ^26^American College of Surgeons: Bangladesh Chapter, Dhaka, Bangladesh; ^27^Bangladesh Health Economist Forum, Dhaka, Bangladesh; ^28^Association of Pediatric Surgeons of Bangladesh (APSB), DMCH, Dhaka, Bangladesh; ^29^Narmada Fertility Centre, Hyderabad, Telangana, India; ^30^International Trauma Anesthesia and Critical Care Society, Stavander, Stavanger, Norway; ^31^Pandit Bhagwat Dayal Sharma Post Graduate Institute of Medical Sciences, Rohtak, Haryana, India; ^32^College of Anaesthesiologists and Intensivists of Sri Lanka, Rajagiriya, Sri Lanka; ^33^Department of Anaesthesia, Perioperative Medicine and Critical Care, AIG Hospitals, Hyderabad, Telangana, India; ^34^Department of Neurosurgery, Northwest School of Medicine, Peshawar, Khyber Pakhtunkhwa, Pakistan; ^35^Army Medical Services, Military Hospital, Thimphu, Bhutan; ^36^Jigme Dorji Wangchuck National Referral Hospital, Thimphu, Bhutan; ^37^Khesar Gyalpo University of Medical Sciences of Bhutan, Thimphu, Bhutan; ^38^LV Prasad Eye Institute, Hyderabad, Telangana, India

**Keywords:** global surgery, SOTA care, South Asia, priorities, health planning, LMICs

## Abstract

South Asia is a demographically crucial, economically aspiring, and socio-culturally diverse region in the world. The region contributes to a large burden of surgically-treatable disease conditions. A large number of people in South Asia cannot access safe and affordable surgical, obstetric, trauma, and anesthesia (SOTA) care when in need. Yet, attention to the region in Global Surgery and Global Health is limited. Here, we assess the status of SOTA care in South Asia. We summarize the evidence on SOTA care indicators and planning. Region-wide, as well as country-specific challenges are highlighted. We also discuss potential directions—initiatives and innovations—toward addressing these challenges. Local partnerships, sustained research and advocacy efforts, and politics can be aligned with evidence-based policymaking and health planning to achieve equitable SOTA care access in the South Asian region under the South Asian Association for Regional Cooperation (SAARC).

## 1 Background

Universal health coverage (UHC) is not possible without equitable access to surgical, obstetric, trauma, and anesthesia (SOTA) care for all. The year 2015 can be considered *Annus Mirabilis* for global SOTA care with the resolution on emergency and essential SOTA care from the World Health Organization (WHO) (1), evidence on disease burden, cost-beneficiality, and cost-effectiveness of essential SOTA care from the Disease Control Priorities Network (DCPN) (2), and evidence on lack of access to SOTA care and developmental gains from scale-up of services from the Lancet Commission on Global Surgery (LCoGS) (3). Since then there has been a global movement for underscoring SOTA care in international and national policy agendas. Assessing systemic and epidemiological indicators related to SOTA care for monitoring and evaluation purposes and introduction and implementation of national SOTA plans are two critical initiatives that multiple countries have invested in. Countries such as Brazil (4), Colombia (5, 6), Mexico and Peru in Latin America (7), Somaliland (8), Madagascar (9), and Uganda (10) in Africa, and Mongolia (11) in East Asia among others around the world have conducted comprehensive high-resolution subnational mapping of indicators for informing policies, while Rwanda, Tanzania, Nigeria, and Zambia among others have committed to national SOTA care plans (12). Despite the global movement, focus on SOTA care in South Asia—one of the world’s most densely populated, culturally diverse, and economically aspirational regions—remains limited.

Here, South Asia refers to the countries in the South Asian Association for Regional Cooperation (SAARC). SAARC includes eight countries: Afghanistan, Bangladesh, Bhutan, India, Maldives, Nepal, Pakistan, and Sri Lanka. The South Asian countries together contribute to 24.1% of the global population (13), 4.2% of the global gross domestic product (GDP) (14), and 25.1% of the global disease burden (15). As of 2023, all other countries except Maldives (upper-middle-income) and Afghanistan (low-income) are lower-middle-income countries (LMICs). The eight countries have several differences across their demographic, socioeconomic, and health-related indicators (16, 17). There are also notable differences in the health systems of these countries (18). All South Asian countries have mixed models for health service delivery engaging public and private sectors at different healthcare levels. The financing is heterogeneous with large portions paid out-of-pocket by patients. However, countries have implemented public financing schemes with varying levels of success (19). Regardless of their differences, these countries share the general trend toward progress on health indicators in the last two decades, limited healthcare financing, and the pervasive issue of within-country inequalities in healthcare provision. Further, different crises have currently impacted healthcare and previous health gains in some South Asian countries (20–24).

This review aims to assess the state of SOTA care in South Asia and provide recommendations for universalizing care in the region. The article proceeds into five main sections that focus on discussing (1) the disease burden and economic burden of surgically avertable conditions, (2) literature on SOTA care indicators for the region and the individual countries, (3) SOTA care relevant policy-making and planning progress, (4) region-wide and country-specific challenges, and (5) potential solutions addressing the challenges.

## 2 Disease and economic burden of surgically avertable diseases

**Figure 1:**
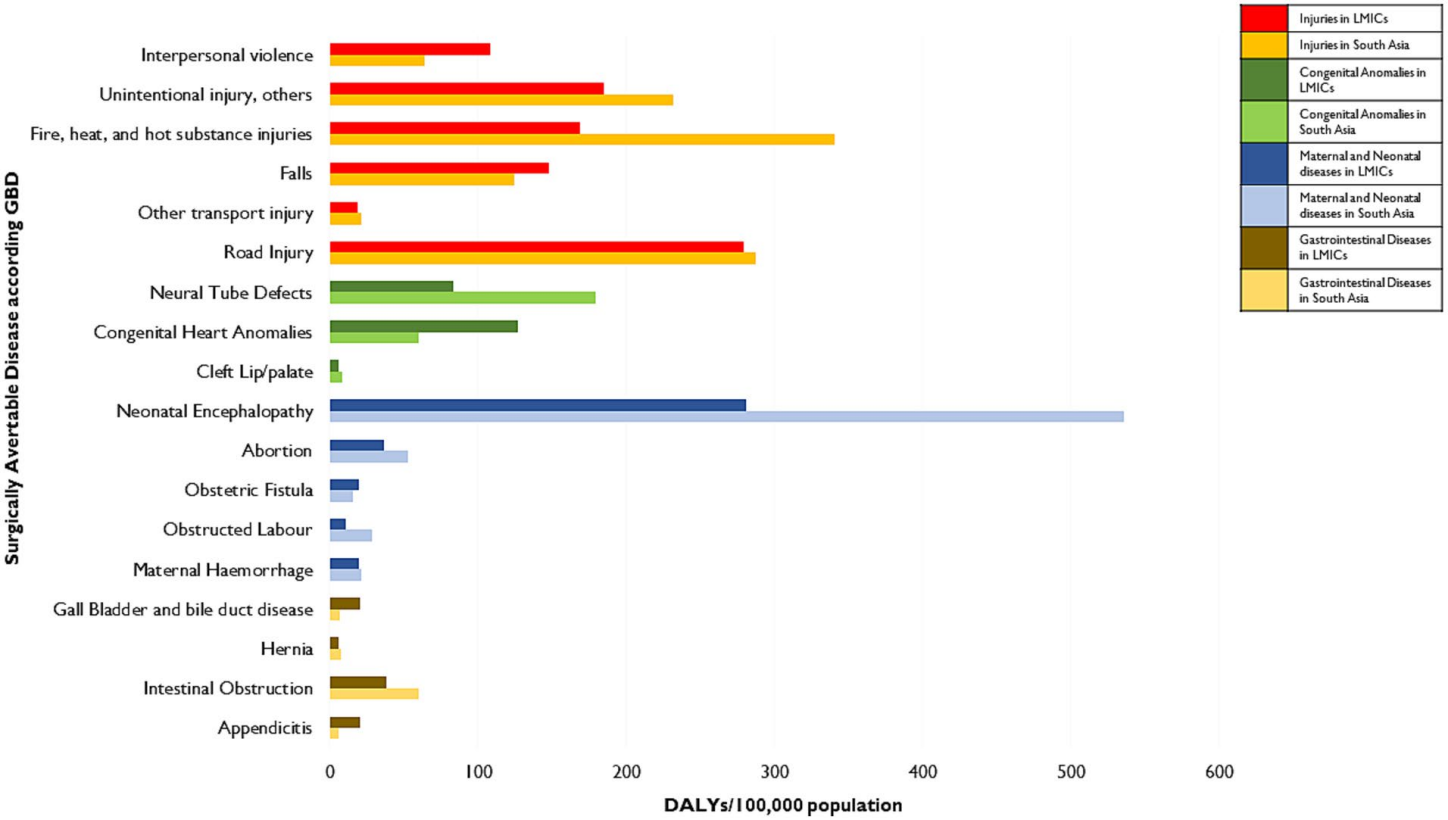
Burden of surgically-avertable diseases in South Asia compared to LMICs. Data has been obtained from studies conducted by Higashi et al. (26–29). Burden is measured in disability-adjusted life-years (DALYs). Unintentional injuries include exposure to mechanical forces, adverse effects of medical treatment, animal contact (venomous/nonvenomous), and unintentional injuries not classified elsewhere in the GBD 2010 Study. Poisoning, drowning, self-harm, and intentional injury, others (including exposure to forces of nature, collective violence, and legal intervention) are not included here.

One of the most critical elements for achieving universal and equitable SOTA care is the monitoring and evaluation of relevant health systems and population health outcomes. An overarching population-level outcome is disease burden measured using disability-adjusted life-years (DALYs) that account for mortality and morbidity. In 2015, DCPN provided a comprehensive assessment of the mortality and morbidity burden due to surgically avertable conditions in low- and middle-income countries (LMICs) (25). Surgically avertable conditions were considered to be the ones that can be provided at first-level hospitals and can potentially improve health outcomes. These included multiple conditions such as obstructed labor, injuries, intra-abdominal emergencies, correctable congenital anomalies, such as clubfoot and cleft lip or palate, symptomatic hernias, cataracts, osteomyelitis, otitis media, etc. The DCPN and subsequent research from Higashi and colleagues noted that South Asian countries contribute to a significant proportion of the burden of surgically treatable diseases among LMICs (25–29). For instance, South Asia contributed 50.46, 32.49, 26.67, and 33.35% of the surgically avertable burden of neonatal and maternal diseases, congenital anomalies, digestive conditions, and injuries. Generally, South Asia had higher avertable disease burden rates, i.e., DALYs per 100,000 population than the overall LMIC rates (Figure 1).

The high avertable disease burden can be partially attributed to limited access to SOTA care. LCoGS defined access to emergency and essential SOTA care as a composite of timeliness, systemic capacity, safety, and affordability. The associated modeling exercise revealed that 5.3 billion people globally lack access to emergency and essential SOTA care (30). Of these, over 1.6 billion or 30.2% of the total people lacking access live in South Asia. This translates to over 98% of the South Asian population lacking access to safe and affordable SOTA care. However, it should be noted that these are modeled approximations that do not represent the differences in access across rural and urban areas, health sectors, population groups, etc. For instance, access in urban areas can be better than that in rural areas partly due to the presence of private SOTA care providers (31).

**Figure 2:**
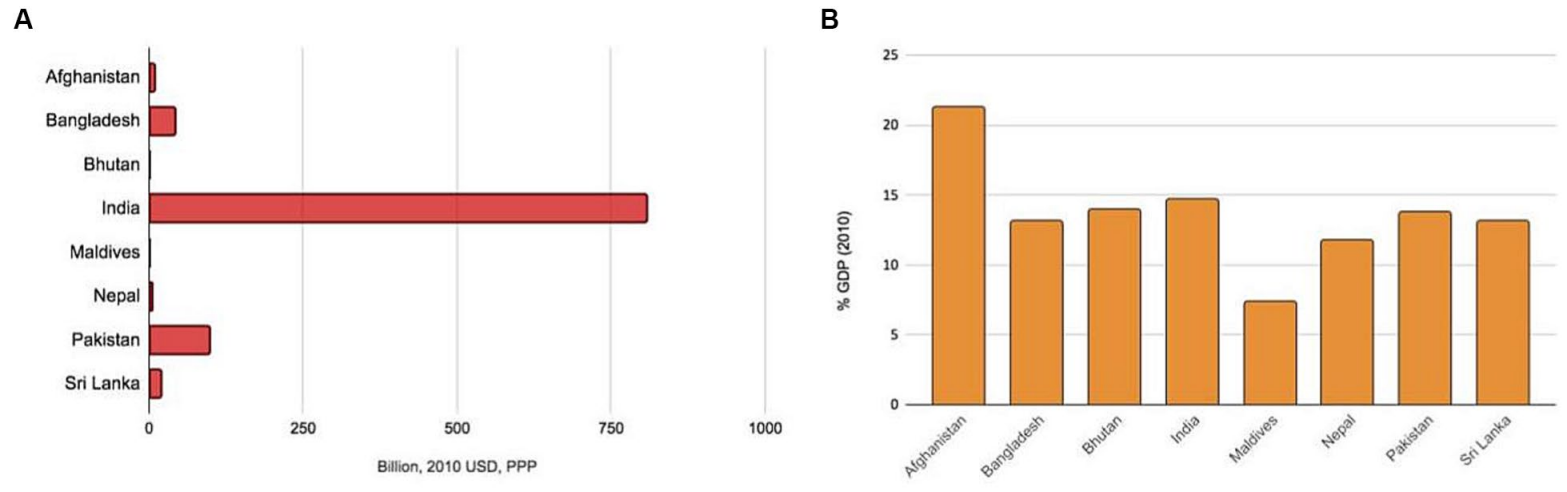
Value of lost economic welfare due to surgically-avertable disease burden in 2010 for South Asian countries as expressed in **(A)** absolute amount in billion 2010 USD PPP and **(B)** % GDP in 2010. Data has been obtained from a previous modeling study by Alkire and colleagues associated with the Lancet Commission on Global Surgery (32). USD, United States dollar; PPP, Purchasing Power Parity; GDP, Gross Domestic Product.

Furthermore, premature mortality and morbidity burden adversely impact the gross domestic product of an economy. This was captured using the value of lost welfare (VLW) in an LCoGS-associated study (32). The VLW approach relies on the value of statistical life-years that capture the long-term losses going beyond accounting for lost workforce productivity, forgone leisure, non-health consumption, etc. The study noted that in the single year—2010, the value of lost welfare for South Asia was about USD 986 billion (2010 PPP), forming 6.8% of the global losses. This value varied across countries from USD 808.49 billion (2010 PPP) for India to USD 0.25 billion (2010 PPP) for Maldives (Figure 2A). The losses also ranged from 21.33% for Afghanistan to 7.41% for the Maldives, expressed as proportions of countries’ gross domestic products (GDPs) (Figure 2B). These losses correspond to only select surgically avertable conditions and the magnitude of losses will only be greater for a more comprehensive enumeration of conditions.

## 3 SOTA care indicators in South Asia

LCoGS suggested six indicators to track surgical care preparedness (timely access and workforce), delivery (volumes and perioperative mortality rates), and impact (catastrophic and impoverishing expenditures) (3). There have also been suggestions for contextualizing LCoGS indicators and adding others that are relevant for trauma and perioperative anesthesia care (33, 34). Collecting data on indicators is critical for monitoring and evaluation which, in turn, is important for evidence-based policymaking and planning. For South Asian countries, research on SOTA care indicators is limited. Data for some of the relevant indicators from international comparisons and those associated with LCoGS are compiled for South Asian countries in Table 1.

**Table 1:** SOTA care indicators for South Asian countries with target achievements marked by green cells.

Country	Surgery indicators							Obstetric indicators				Anesthesia indicators		Trauma indicators	
	LCoGS I (35)	LCoGS II (36)	LCoGS III (37)	LCoGS IV	LCoGS V (38)	LCoGS VI (38)	SPI (39)	Met need for EmOC (40)	Cesarean section rates (41)	OBGYN density (36)	MMR (42)	Anesthetist density (36)	AOC index (43)	VNRD (44)	Blood safety protocol (44)
Afghanistan	–	0.03	229	–	64.3	92.8	77	20	6.6	–	638	0.03	0.33	2.26	Yes
Bangladesh	96.6	3.02	162	–	48.5	73.6	87.2	9	32.7	0.5	173	0.8	0.48	1.04	Yes
Bhutan	–	2.87	2690	–	18.1	37.7	–	49	12.4	1.8	183	0.4	0.15	7.21	Yes
India	–	6.82	904	–	36.5	59.6	92.4	27	17.2	2.4	145	1.6	0.31	6.88	Yes
Maldives	–	6.73	558	–	–	23.2	–	–	40.0	3.8	53	0.6	0.44	4.77	No
Nepal	–	2.96	209	–	42.7	74.8	85.7	19	9.0	1.2	186	0.7	0.30	7.92	Yes
Pakistan	–	5.53	423	–	40	75.2	81.7	9	22.3	2.8	140	1.5	0.02	1.87	Yes
Sri Lanka	99.9	3.03	2920	–	16	58	81.5	75	33.2	0.6	36	0.6	3.49	20.80	Yes

I, timely geographic access, target—80% population (3); II, SAO Workforce, target—20 SAO per 100,000 people (45); III, annual surgical rate, target—5000 per 100,000 people (46); IV, POMR, target—100% tracking (3); V, impoverishing health expenditure, target—0% surgery-seekers face IHE (3); VI, catastrophic health expenditure, target—0% surgery-seekers face CHE (3); SPI, Surgical Preparedness Index—mean value; Met Need for EmOC, Met Need for Emergency Obstetric Care (latest nationwide numbers for each country were taken. In case nationwide data was unavailable, latest study with maximum number of districts was considered), target—100% (47); cesarean section rates (latest cesarean section rate of each country was considered), target—10-15% (48); MMR, maternal mortality rate (2017 data for all the countries was considered), target—< 70 per 100,000 live births (49); AOC, adequacy of prescription opioid consumption based on normative threshold, target—100 (50); VNRD, voluntary non-remunerated donations per 1,000 people using population counts from World Bank (2018), target—15 units per 1000 people per year (3); blood safety protocols, Present of national standards for the collection, testing, processing, storage, and distribution of blood and blood components. The values are equal to or exceed the recommended targets are mentioned in green color shade.

There is limited national-level data on timely access to surgical care (51). The available data for Sri Lanka and Bangladesh suggests that they have crossed the LCoGS target of 80% population within 2 h of travel time from a facility providing bellwether procedures (52). For the SAO workforce, recent comparable data are not readily available but that collected and imputed during LCoGS suggests that all South Asian countries fall short of the target of 20 surgery, anesthesia, and obstetric (SAO) specialists per 100,000 population (36). Modeled estimates for population-level surgical volumes depict deficits in all countries compared to the target of 5,000 surgeries per 100,000 people per year (37). Further, models also suggest that at the current rate of scale-up, no country in South Asia would achieve the target rate before 2035 (53). While a systematic review provides a compilation of POMR studies in LMICs (54), data on South Asian countries are scattered. More importantly, there are no standard national perioperative mortality rate registries across the eight countries. Estimates for catastrophic and expenditures based on cesarean section rates depict that risk protection against catastrophic and impoverishing expenses due to seeking surgery when needed is limited and does not meet the aspirational target of 100% protection coverage set by LCoGS (Table 1) (38, 55). There have been some recent preliminary efforts for subnational data collection and/or estimation of LCoGS indicators for India (31, 56–59) and Pakistan (60, 61), but high-resolution subnational data remain largely missing for other countries.

Going beyond LCoGS, the GlobalSurg and COVIDSurg Collaboratives introduced and validated a hospital-level elective surgery assessment named surgical preparedness index (SPI) which is a composite of 23 variables capturing facilities and consumables, staffing, prioritization, and systems (39). Bangladesh, India, and Nepal had SPI scores above the global average of 84.5. Bhutan and Maldives did not have data. For obstetric indicators, we used cesarean section rates, obstetrics and gynecology (OBGYN) specialist density, met need of emergency obstetric care (EmOC), and maternal mortality rate (MMR). The WHO recommends that cesarean sections should form 10–15% of deliveries for a country to lower its MMR (48). Across South Asia, countries face the dual challenge of inadequate cesarean section rates in some places and excess cesarean section in others. For instance, only Bhutan has a rate that falls within the WHO recommended range. Afghanistan and Nepal fall below the threshold while other countries exceed the recommended cesarean section rates (41). Excess cesarean sections are a well-documented public health problem in South Asia (62). OBGYN density is poor in all South Asian countries (36). Except for the Maldives and Sri Lanka, all other countries also fall behind in achieving the target of reducing MMR to 70 per 100,000 live births by 2030 (49). Except for Sri Lanka, all the other countries have less than 50% of EmOC met need (40).

National data on the trauma care workforce and national registries on trauma surgical volumes and mortality are severely limited, though there have been recent studies conducted in parts of India and Sri Lanka (63–65). Adequate and safe supply of blood for transfusions is an important component of trauma care for which some data is available. LCoGS noted that 15 units per 1,000 persons per year is an adequate standard for a voluntary blood donation rate (3). A cross-country comparison of blood banking conducted by WHO reveals that among the South Asian countries, only Sri Lanka satisfies this threshold (44). On the positive side, except Maldives, all other South Asian countries have national standards in place for ensuring safe blood transfusions. For anesthesia care, anesthetist density is limited across all countries (36). Another important indicator is an adequate supply of analgesics for perioperative pain management which can be captured using the scaled index called adequacy of opioid consumption (AOC) (50). Estimation of the AOC index reveals that the values for all South Asian countries are below one compared to the target of 100 (43).

## 4 SOTA care planning in South Asia

The World Health Assembly Resolution 68.15, which noted the importance of emergency and essential SOTA care for UHC, was signed by all South Asian countries (1). However, greater policy attention and political will for specific investments directed toward SOTA care are needed. African countries such as Rwanda, Tanzania, Zambia, etc. have formulated National Surgical, Obstetric, and Anesthesia Plans (NSOAPs) that provide pathways toward improving surgical care by strengthening surgical systems in alignment with the countries’ national health policies and plans (12).

In South Asia, there has been some development concerning SOTA care planning. The Ministry of National Health Services, Regulation and Coordination in Pakistan has drafted a National Surgical Care Vision 2025 with the involvement of local and national partners including health service delivery organizations, individual practitioners (specialist surgeons, OBGYNs, anesthetists, and pediatric surgeons) and health planners and support from international organizations such as WHO (66). In terms of policy framing, it is in line with Pakistan’s National Health Vision 2016–2025. The National Surgical Care Vision 2025 accounts for the federal-provincial health system structure in Pakistan and has proposed the development of detailed Provincial Surgical, Obstetric, and Anesthesia Plans to cater to local needs and demands. With support from the United Nations Institute for Training and Research and several other global partners, the Ministry of Health and Population in Nepal has also initiated its NSOAP development process by conducting high-level stakeholder orientation meetings (67).

In the case of India, after LCoGS, the National Surgical Forum was conducted in 2016 where important stakeholders discussed priorities and potential implementation opportunities for improving SOTA care in India (68). The Karad Consensus Statement drafted by the Association of Rural Surgeons of India was signed by multiple partners (69). Additionally, under the implementing-LCoGS-India initiative centers for excellence were started (70). All these efforts have identified the poor and unsafe blood supply infrastructure in the country, low rural SOTA workforce availability marred by limited training and unsustained partnerships, as well as lack of needs assessment, relevant targets, and surgical innovations scaleup for rural India as significant gaps. Importantly, India has a National Programme for Prevention & Management of Trauma & Burn Injuries aimed at capacity building (infrastructure, human resources, equipment, etc.) in public hospitals for quality trauma care provision (71). However, the scheme was valid only till March 2020. A dedicated SOTA care plan is still lacking for India and its states (72). Further, there is also some evidence that SOTA care has had limited prioritization in national policies and programs across the last seven decades (73). Of note, it is also crucial that decision-making in health-related matters is driven by evidence and not just political priority.

Sri Lanka has a “National Policy and Strategic Framework on Injury Prevention and Management” that includes pre-hospital and emergency trauma care under secondary prevention and more recently a “Multi-sectoral Strategic Action Plan on Injury Prevention and Management 2021–2025” that envisions developing clinical management guidelines for trauma care at all levels of care, establishing a trauma cluster care system, ensuring adequate capacity and training of staff for trauma patients, and collecting appropriate data for assessment (74, 75). To our knowledge, there has not been any development toward national SOTA care planning in other South Asian countries. Urgent attention, committed investments, and sustained efforts are necessary for equitable and universal SOTA care. This can be achieved through local collaborations within the region and shared learning under SAARC.

## 5 Current challenges

### 5.1 Suboptimal distribution, utilization, and quality of services

Suboptimal distribution and utilization of services especially at the primary and secondary levels of care enhance access disparities. On the supply side, resource allocation, including staffing, based on one-size-fits-all standards leads to wastage of resources at some SOTA care facilities while shortage at others. On the utilization side, secondary-level care facilities remain under-utilized while tertiary hospitals are over-burdened by patients who bypass the referral system in search of assurance for better SOTA care. While the limited geographical access to SOTA care facilities might be high in some South Asian countries as noted before (see Table 1), quality of care needs improvement on several fronts. The lapses in quality can be attributed to inadequate human resources and limited financing for the development and maintenance of SOTA care facilities (3, 76).

### 5.2 Inequitable obstetric care—cesarean section disparities

Access to safe and affordable obstetric care, including cesarean sections, is highly inequitable. While the national aggregate numbers show excess cesarean section rates compared to the WHO threshold for multiple South Asian countries, it is noteworthy that several factors lead to large within-country differences in cesarean section rates (77–79). Cesarean sections are frequent in urban private facilities among older, educated, and financially independent women (77). However, access to cesarean sections in rural regions is limited. For instance, using the rate values (as % institutional deliveries), when we calculate rural-to-urban ratios for cesarean sections, they come out to be 0.87, 0.54, 0.82, and 0.53 for Bangladesh, India, Maldives, and Nepal, respectively (78). Cesarean section rates are also known to be different across public and private health sectors. When we calculate private-to-public ratios using the values for the cesarean section rates (as % institutional deliveries) for Bangladesh, India, and rural Nepal, we get 2.52, 1.00, and 2.14, respectively (80). Seeking healthcare in private healthcare facilities is often beyond the reach of women from the lower socioeconomic strata, which in turn points to disparities in access to cesarean sections. More broadly, for a large section of women in South Asia safe, affordable, and rights-based obstetric care remains beyond reach (81, 82).

### 5.3 Limited SOTA care in rural areas

SOTA care delivery in rural and remote areas is a challenge faced throughout the region. Lack of infrastructure and limited workforce availability are major impediments to service delivery (31, 69, 83–85). Infrastructural challenges can be wide-ranging from poor road conditions limiting access to the hospitals, frequent power outages, limited maintenance of operating rooms, inadequate surgical equipment, insufficient and irregular supply of blood products and drugs, lack of lodging facilities for patients and their caregivers during the perioperative period, etc. Innovations by rural SOTA care experts can mitigate several issues. For instance, the last decade has seen massive progress in the development of minimally invasive surgical equipment, including gasless laparoscopic systems tailored specifically to the needs and contingencies of rural South Asia (86–88). Similarly, innovative alternatives for surgical techniques have been tested that can ensure safe SOTA care delivery at reduced costs to rural patients (89–91). However, a consistent investment in SOTA care innovations is missing and additional pathways for scale-up and integration of such innovations are required.

While the South Asian region has low SAO workforce densities (see Table 1), however, these numbers drop further for rural areas. Among the workforce personnel, there are fewer anesthetists than surgeons, even at district-level hospitals, which limits the delivery of emergency and essential surgeries (85). Further, those working in rural and remote might require additional training and competency building to adequately deal with uncertain and challenging conditions (e.g., frequent power outages, working without appropriate equipment, etc.). However, specialty programs providing such training are limited (92–94). Additionally, rural and remote areas are severely underrepresented in research which necessitates data collection that can be used to assess the efficacy and safety of interventions and innovations being undertaken at rural hospitals.

### 5.4 Workforce migration

Multiple South Asian countries suffer from the “brain drain” due to the emigration of SAO specialists, which leads to the weakening of the SOTA care systems (95). The proportion of surgical specialists trained in South Asia who practice abroad ranges from 3.9% for Nepal to 61.1% for Sri Lanka (96). There are multiple reasons for emigration with opportunities for better quality training, prospects for employment, and improvement in socioeconomic status being some of the main ones (97). These emigration rates are comparable to those among general physicians and other specialists. Hence, data on physician emigration can be a useful proxy. In the United Kingdom alone, physicians trained in India, Pakistan, and Sri Lanka contribute to 24.5% of the total physicians (98). Overall, the physician brain drain costs South Asian countries about 5.2 billion USD annually (99).

Beyond specialists and physicians, the brain drain is also prevalent among nurses who play a critical role in delivering SOTA care services. For instance, the countries accounting for the largest shortages (in absolute terms) in 2018 included Bangladesh, India, and Pakistan. From April to September 2021, more than 10,000 new international nurses were registered in the UK, of these over 4,500 were from India (100).

### 5.5 Burden of road traffic injuries and limited trauma care

Rapid growth in the past years, high urban population density, and limited planning in countries such as India and Sri Lanka have presented them with a growing burden of road traffic injuries (RTI) (17, 101). India and Sri Lanka together contribute to 36% of global RTI incidence and 18% of mortality (102). Further, the RTI burden is also associated with a greater financial burden captured by higher out-of-pocket spending and catastrophic health expenses in households with RTIs compared to those without an RTI event (103). This points to a clear need to invest in upscaling trauma care systems including the blood transfusion capabilities. Delays in access due to communication gaps in prehospital care, limited training for basic clinical care within hospitals, and lack of trauma data systems as well as necessary administrative support are major systemic barriers to trauma care for RTI patients in India (104). Recently, there have been more efforts to set up hospital-based trauma registries in India and Sri Lanka (63–65). However, ensuring continued functioning and high quality of such registries is a challenge due to limited policy attention to SOTA care and funding constraints.

India has seen progress in the last few decades for emergency and trauma care with public-private service delivery models such as “108 emergency ambulances” operated by the EMRI Green Health Services formerly known as the GVK EMRI (105) and neurotrauma management including the comprehensive guidelines from the Neurotrauma Society of India for integrated prehospital, hospital, and rehabilitative care of traumatic brain injury (106). Similarly, Sri Lanka has witnessed calls for establishing a National Trauma System that can optimize patient referrals and reduce injury-related mortality (107). India and Sri Lanka have policies and programs directed toward trauma care but a comprehensive assessment of their implementation remains to be seen.

### 5.6 Poor access to anesthesia and pain management

India and Sri Lanka also share some challenges related to anesthesia care. First, most anesthetists work as consultants working between the public and private healthcare sectors (108, 109). This means that their availability at a given health facility cannot be assured which impacts public SOTA care systems (85). Second, there is limited access to perioperative pain management (see AOC values in Table 1). The limited number of anesthesiologists who can prescribe such medication, high costs for patients, and the complex regulatory framework that intends to avoid opioid misuse but simultaneously limit access to prescription pain medication, are some of the factors that make pain management challenging. The economic crisis in Sri Lanka is further expected to exacerbate these challenges. Over 65% of the anesthetists trained in Sri Lanka typically emigrate to HICs (110). This emigration can rise further as consultant anesthetists try to look for stable work opportunities and better professional lives. Access to pain medications can go down due to supply chain disruptions (111). Both these threats can reduce the quality of care in the country despite organized efforts of Sri Lankan anesthetists to make ends meet in a strained health system (112). Most importantly, such challenges, while prevalent, are not given the deserved attention in the broader global SOTA care discourse.

### 5.7 Neglect toward pediatric care

South Asian countries constitute some of the largest pediatric (under 18) populations globally with a high burden of amenable pediatric mortality (113), yet pediatric SOTA care capacity is limited (114, 115). The critical threshold for needed pediatric surgical workforce density per 100,000 children under the age of 15 years is found to be 0.37 (116). While Maldives has a density above this threshold, Bangladesh, India, and Pakistan do not (116, 117). Data on other South Asian countries is limited. A shortage of pediatric SOTA care workforce is associated with greater neonatal, infant, and under-5 child mortality (115). Moreover, data from India depicts that pediatric surgery contributes to only 0.7% of the total subspecialty training spots (118). This depicts a lack of training capacity that can contribute to the continuation of workforce shortages in the future. Similarly, data from Pakistan depicts an unmet need for pediatric surgery due to a lack of trained workforce due to limited capacity and low quality of training (119, 120). Nepal noted limited services, lack of money and time, and lack of acceptability toward surgical care providers as reasons for unmet pediatric surgery needs (121).

### 5.8 Limited attention to allied professionals in SOTA care

The role of nurses and midwives in improving access to quality services, especially among the hard-to-reach populations, often goes unappreciated in global SOTA care discourse. South Asia faces a large burden of maternal mortality due to hemorrhage, eclampsia, obstructed labor, sepsis, and unsafe abortions among other reasons that can be prevented through timely access to cost-effective interventions (122). Scaling up well-trained nurses and midwives to ensure complete skilled birth attendance coverage is important. In rural and remote areas lacking access to trained specialists and physicians and equipment and technologies, experienced nurses and midwives handle complications during deliveries. While South Asian countries have observed progress toward achieving international nursing and midwifery standards, there is a long road ahead (123).

### 5.9 Impact of COVID on SOTA care

Similar to other parts of the world, SOTA care in South Asia suffered from disruptions due to the COVID-19 pandemic. The disease spread and the movement restrictions aimed to curb it, stalled service delivery and adversely impacted training. For instance, early on in the pandemic, volumes of emergency surgery were reduced to lockdowns, elective surgeries, and outpatient appointments were canceled followed by delays (124). At the Phuentsholing General Hospital in Bhutan, cesarean section deliveries dropped by 6.6% and gynecological surgeries dropped to 13.9% of the total gynecological services from 20.6% during the pandemic years (2020–21) compared to 2019 (125). Across cancer, cardiovascular, respiratory, and other conditions, surgical treatments during COVID-19 (2020–21) were reduced by 4 to 97% across different studies for India compared to 2019 and previous years (126). In Nepal, all neurosurgery centers postponed elective surgeries and shut down outpatient care early on in the pandemic (127). Major reductions in training hours and capacity were recorded for obstetrics and gynecology residency training programs in India and general surgical residency in Pakistan among others (128, 129). These point to the need for including SOTA care in public health emergency response. Further, there is also a need to expand the notion of emergencies beyond epidemics or pandemics to include mass casualties and climate emergencies (130, 131).

### 5.10 Lack of need-based policy-making

Policymaking related to SOTA care needs to be determined based on patient needs. For instance, pain management significantly burdens surgical patients. About 20–25% of the global population experiences chronic pain but management in South Asia is faced with barriers due to cultural beliefs, physician education, infrequent use of standardized pain management tools, and healthcare infrastructure (132). The rest of the above-mentioned challenges also call for tailored advocacy. Hence, any policy and planning efforts should include this and other such issues based on the lived experiences of the patients and suggestions of the providers.

### 5.11 Some country-specific challenges

Given the diversity in the region, beyond those noted above, some challenges are more pronounced in some places than others. For instance, political instability and the COVID-19 pandemic have contributed to the extant challenge of high maternal mortality in Afghanistan. This can be, in part, attributed to the limited availability of obstetric surgeons, nurses, and other healthcare workers (133). Bhutan has been hiring surgeons and anesthetists from other countries. Currently, training programs for physicians and surgical specialists are limited (134), which forces the country to recruit and fund medical students in other countries who are willing to commit to serving in Bhutan post-training. In Nepal, accessibility, affordability, and acceptability (lack of trust in providers or fear of healthcare seeking) are major barriers to SOTA care (135, 136). National projections suggest that 2.4 million residents in Nepal may not get care when they need it (137). Further, differences in capacity and quality of care, as well as training of surgical specialists, play an important role in within-country variations in SOTA care provision (138).

## 6 Potential directions

Achieving equitable SOTA care in South Asia is dependent on local partnerships that enable the free exchange of knowledge, resource mobilization, and priority-setting. Achieving equitable SOTA care requires collaborative and sustainable efforts in various directions. Though not exhaustive, we list some important directions here.

### 6.1 Enhancing SOTA care research efforts

Research efforts for data on SOTA care indicators are needed for all countries. Such a research program should ensure the following:

(a) Identification of disparities. Emphasis should be on high-resolution subnational estimates instead of aggregate national numbers. Further, disaggregated data for rural and urban regions, and types of healthcare sectors (public, nonprofit/trust/faith-based, and for-profit private) are essential for the equitable distribution of resources.
(b) Focus on under-resourced settings. It is crucial to orient research efforts and resources toward rural and remote areas as well as underserved populations to quantify the needs and test effective interventions.
(c) Ownership by and opportunities for local research investigators. The research has to be locally owned and governed and not too reliant on limited-term external funding.
(d) Ensuring equity within the investigators in terms of gender, institutional portfolio, regions, etc. is further important to ensure the decentralization of research efforts.
(e) Priority setting by local interested/affected parties. While the work can be facilitated through international collaborations including high-income country academic institutions and funders, the research agenda should be set by those working for and affected by SOTA care issues including caregivers, patients, advocates, and policymakers. Diversity in decision-making over priorities might be challenging to manage but it can help better align limited resources toward feasible and actionable goals.
(f) Setting contextually relevant targets. While LCoGS, WHO, and other international and intergovernmental efforts have provided targets for various SOTA care indicators, it is critical to rethink and revise them according to the regional/local context. Participatory research for deciding contextually relevant targets should consider the trade-off between aspirational and implementable targets.
(g) Evidence for targeted interventions. The research needs to go beyond the measurement of problems and should be oriented toward potential solutions. Testing of efficacy and safety, effectiveness, cost-effectiveness, and implementation feasibility of different interventions is central. Further, evidence synthesis for new interventions and tailoring existing interventions for specific populations, geographies, etc. is critical for success given the within and between-country diversity in South Asia.
(h) Leveraging existing data systems. Research should focus on auditing, collating, and utilizing extant data sources and systems for SOTA care indicators before initiating primary data collection given that the latter is resource-intensive (72). Resources can thus be optimally allocated to epidemiological and interventional research.
(i) Working toward absorptive capacity for integration of SOTA care indicators. Rather than introducing new systems for parallel data collection dedicated to SOTA care, these indicators should be integrated into current health management and information systems and demographic surveys for sustained monitoring and evaluation (139).

### 6.2 Training and retaining high-quality SOTA care workforce

High-quality training of surgeons, obstetricians, anesthetists, nurses, and allied professionals as well as timely scale-up of the SOTA care workforce needs to consider the following:

(a) Investing in increasing residency and fellowship level training spots for the next generation of SAO specialists is the key. The scale-up should happen at an adequate rate/level in a target-oriented manner. For instance, to achieve the target density of 20 SAO specialists per 100,000 population by 2030, Bangladesh, India, and Pakistan would need 40,288, 291,824, and 47,710 more SAO specialists compared to 2015 (140). Additionally, the training spots should be equitably distributed (141).
(b) Retaining existing SAO specialists and ensuring their distribution across different parts of the country should be prioritized. However, there has to be a major policy change in the context of curbing the “brain drain.” Punitive policies need to be replaced with those that focus on strengthening health systems to ensure better working environments and behavioral modifications as well as incentives for SAO specialists to serve in their countries of origin (85, 142). Opportunities for professional growth and better environment and security for families of SAO specialists can help in retention. It is also important to integrate and retain the nursing and midwifery workforce into SOTA care as they contribute to health system strengthening.
(c) Changes in training to ensure high-quality service delivery and good patient outcomes are necessary. Rural surgery residency, fellowship, and short-term programs that cut across subspecialties to equip trainees with skills to operate under resource constraints should be expanded. For instance, promoted by the Association of Rural Surgeons of India, the National Board Examination offers a 3-year DNB in Rural Surgery (postgraduate diploma program) (143). The COVID-19 pandemic brought out some innovative and resilient methods of training that need to be integrated into the system. For instance, telementoring, hybrid models of learning, and a greater focus on the well-being of trainees as observed in Pakistan could be tested in other countries for potential scale-up (129, 144).
(d) Participating in shared training opportunities including exchange programs and rotations is critical for South Asian countries to ensure good quality SAO workforce. Effective implementation of such multi-country or exchange programs would require competency-based training, standardization of curricula, independent quality assessments, and uniform accreditation standards. Such efforts can particularly benefit countries with limited programs including Bhutan and Nepal. In the long run, they can promote pathways for multi-country clinical licenses and intra-regional SAO specialist need-based movement to improve equitable distribution.
(e) Providing opportunities for research and advocacy skills learning for those who are inclined toward these is also necessary as these skills are different yet can be equally important as clinical training for revolutionizing SOTA care access. While integrating cursory introduction to these in routine training is important, such opportunities should be more trainee-led. For instance, trainee-led collaborations (e.g., PakSurg) and peer-led student research interest groups or networks that are aligned with the country’s research needs have seen some initial success in Pakistan (145, 146). There have been proposals for advocacy fellowships that would train residents in policy-brief and op-ed writing, public speaking, traditional and social media advocacy, activism, story-telling, etc. (147)
(f) Training of allied (non-specialist) professionals who are currently or can be potentially involved in delivering SOTA care services should be urgently considered. There is accumulating evidence on the need and practice of task- shifting and sharing (TSS) in global SOTA care (148). Training of allied professionals for specific surgical or anesthesia management skills has been gaining momentum in some South Asian countries with trials in Afghanistan, Bhutan, India, and Nepal (149). TSS initiatives can be classified into those where some tasks performed by specialists are shifted to/shared by adequately trained non-specialist physicians and those where the tasks performed by specialist or non-specialist physicians are shifted to/shared by other health professionals including nurses, midwives, technicians, etc.

It is important to note that beyond evidence on effectiveness, for successful introduction and implementation of TSS initiatives at programmatic scales, understanding acceptability from involved parties (e.g., SAO specialists) is crucial. There has been greater acceptance of task-sharing than task-shifting (150). Additionally, the specialist to non-specialist TSS has faced less resistance from SAO specialists (149). Hence, interventions establishing the effectiveness of TSS should be accompanied by interventions that improve their acceptability among the existing SAO workforce. Regulatory barriers also need to be navigated for successful implementation as several South Asian countries currently allow only SAO specialists to independently perform several procedures. Perhaps, for South Asia, there needs to be some rethinking around the traditional approaches used in TSS. Ultimately, the broader idea of team building and optimization needs to be focused on in this discourse.

### 6.3 Harnessing technical innovations in SOTA care

South Asian countries can leverage technical innovations—equipment, technologies, and procedures—to mitigate the challenges associated with resource constraints and access disparities. Over the last three decades, there have been several instances of low-cost surgical equipment designed with a human-centered approach and safe alternative procedures/techniques born out of the need that have enhanced access to SOTA care in rural and remote areas. For instance, gasless laparoscopy as a part of the broader suite of minimally invasive surgery has improved access to care, reduced costs, and ensured patient safety for basic procedures such as cholecystectomy, appendectomy, etc. (86, 151). Growing uptake of telemedicine and digital health in South Asia also has a role in improving SOTA care access. Telemedicine including phone calls, video chat, and WhatsApp-like services is increasingly used for perioperative patient consultations, postoperative follow-up, and provider-to-provider communications. Digital tools are also being used for learning at a distance, improving access to education and training (152). Systemic propagation of such innovations needs: (a) dedicated funding mechanisms for early-on development, (b) fast-tracked pathways for testing and establishing efficacy and safety, (c) well-structured regulatory environments for mainstream integration, and (d) implementation grants specifically targeted toward their uptake in low-resource settings that can benefit the most. It is critical to note that for achieving health equity, cost-effective technical innovations (e.g., gasless laparoscopy) that can potentially cater to common problems of large populations needing emergency and essential SOTA care and must be prioritized over costly innovations (e.g., robot-assisted surgeries) (153) that can be housed only in urban multispecialty tertiary centers that can be accessed by a select few.

### 6.4 Promoting healthcare tourism across South Asia

Promoting healthcare tourism in the context of SOTA care across South Asian countries can benefit patients while building regional cooperation and solidarity in the long run. Healthcare tourism is popular in the region with India as a major hub for receiving patients from other South Asian countries. For instance, 53.3% of medical tourists coming to India are from South Asian countries while these countries contribute to only 17.2% of general tourists, depicting a clear priority for health seeking (154). A large chunk of healthcare tourists visit private sector hospitals in India for SOTA care treatments related to orthopedic and trauma surgery, surgical eye care, plastic surgeries, cancer care, etc. (155). While variations exist across sender South Asian countries the overall trend in healthcare tourism has been increasing over years thereby establishing the need for expanding the capacity to serve such patients. Regional tourism makes SOTA care accessible but seeking care in the private sector can put a financial burden on patients, especially those arriving from lower socioeconomic strata of low-income countries. Additionally, logistic and legal hurdles involved in migration can deter those in need of care.

There are multiple ways to improve SOTA care access for South Asian healthcare tourists: (a) Ensuring that SOTA care tourism is not limited to those who can afford it but includes everyone in need is most important. The SAARC member nations can initiate a shared financing mechanism for supporting SOTA care tourists across countries. Such financing can learn from and add to the current efforts toward universal healthcare coverage in South Asian countries. For instance, India recently expanded its government-funded healthcare insurance to cover the bottom 40% of the country’s population under the Pradhan Mantri Jan Arogya Yojana (PMJAY). While ambitious in its vision and implementation, there have been notable limitations in the equitable uptake of the scheme that need to be rectified for target coverage achievement (156). (b) Accreditation of SOTA care facilities and standardization can ensure high-quality service delivery (157). (c) Resources for healthcare tourism should be based on demand patterns that depict investments in building SOTA care capacity. (d) Continuing to cut down red tape and easing up visa processes for healthcare tourists can further improve access (158).

### 6.5 Scaling up model (exemplary) SOTA care systems

Learning from and adapting exemplary or model SOTA care delivery systems and initiatives for scale-up can have large gains with some assurance about implementation feasibility. In 2015, LCoGS initiated cataloging such models including Jan Swasthya Sahyog in India and Indus Hospital in Pakistan among others (159, 160). Cataloging more such models operational across South Asia is essential. Beyond compiling high-level evidence, the catalog can act as an important resource to demonstrate to decision-makers reliability and local stewardship regarding universal and equitable SOTA care. Examples of models include a collaborative trauma care initiative for managing road traffic injuries in Nepal (138), quality improvement model solving infrastructural challenges in the maternity ward of the national referral hospital in Bhutan (161), and a sustainable and efficient model for low-cost cataract surgery in India among others (162). The catalog can be further expanded to models beyond South Asia to include countries and settings that can promote Global South–South learning.

### 6.6 Boosting advocacy efforts

Globally, effective advocacy has played a major role in reducing the neglect of SOTA care and ensuring its inclusion in intergovernmental resolutions and policy agenda. However, only local advocacy efforts can make a true difference. Advocacy for attending to and investing in SOTA care is based on multiple complementary arguments. Ensuring SOTA care for all is embedded in countries’ commitment to human rights (163). Hence, by working on SOTA care access, policymakers and political leaders can affirm their commitment to the rights of their citizens. Expanding SOTA care has a major role in countries achieving universal health coverage (164). Further, SOTA care access is also tied to multiple SDGs beyond health (165). Essential and emergency SOTA care interventions are as cost-effective as immunization and other common healthcare interventions (166). Not investing in SOTA care will lead to health losses for South Asian countries’ populations (see avertable burden in Figure 1) (25–29) and financial losses for the aspiring economies (see welfare losses in Figure 2) (32).

SOTA care advocates have opportunities to learn from the advocacy movements for universal health coverage (UHC) and primary health care (PHC). For instance, understanding the political prerequisites and governance nuances has been critical for UHC advocacy (167). While aligning with demographic transitions, integrating technological innovations, formulating regional strategies, focusing on inequalities, exploring alternative financing models, involving community-level workers, etc. have been important for the prioritization of the PHC movement in Southeast Asia (168).

For success, advocates for SOTA care in South Asia should be aware of and act on the following:

(a) Speaking the language of policymakers. Health policymaking is complex with no set model and can differ across countries and contexts. Advocates must align SOTA care with the priorities of policymakers and present evidence in ways that are acceptable or appreciable to those involved in the decision-making process. For example, this involves assimilating academic studies into simpler policy briefs.
(b) Understanding local power sharing. Stakeholder mapping is a crucial tool for understanding the roles held by different players and how they influence each other in a formal or informal capacity. Further, in several South Asian countries, relying on personal and informal connections beyond formal professional collaborations can help further the cause. Hence, local SOTA care advocates from grassroots service delivery organizations, tertiary care centers, academic institutes, think tanks, etc. who have established connections have a major role to play.
(c) Advocates should focus on specific asks supported by evidence and consensus. In the case of South Asia, these can include monitoring and evaluation of SOTA care indicators, integration of these indicators in national targets and policies, advocating at the intergovernmental level for integration of SOTA care among international targets, development and implementation of national and/or subnational SOTA plans, inclusion of SOTA care issues horizontally across other health, environment, economic welfare, and social development policies and programs, and sustained financing for SOTA care scale-up.
(d) The SOTA care advocacy community should be more inclusive and expand beyond SAO specialists. Successful advocacy is dependent on involving various interested and affected parties such as non-specialist SOTA care professionals (nurses, midwives, etc.), non-SOTA health workers (community health workers, pharmacists, etc.), researchers, health-technology entrepreneurs, journalists, funders, and most importantly patients. These parties bring diverse views and skills and can increase the size of the audience and enhance engagement. Bringing out patient voices that share lived experiences about lack of timely geographical access, facing low-quality care, and suffering from financial distress can add to the appeal for political leadership beyond the evidence.

### 6.7 Involving SAARC Secretariat

Bringing SOTA care to the SAARC Secretariat’s initiatives is critical. Previously, SAARC had recognized bodies relevant to SOTA care including the SAARC Surgical Care Society headquartered in Colombo, Sri Lanka, which had a valid recognition till 2017, the Federation of Association of Pediatric Surgeons of SAARC Countries headquartered in Chittagong, Bangladesh, valid till 2019, and SAARC Academy of Ophthalmology headquartered in New Delhi, India, valid till 2017 (169).

The Secretariat should establish and sustain a dedicated SAARC Center for SOTA Care to lead future initiatives and collaborations in all domains of SOTA care. This can be done in multiple ways that have positive implications for SAARC Areas of Cooperation as detailed in Table 2. The Center can learn from the successful functioning and follow the governance structure of the SAARC TB and HIV/AIDS Centre (171). Ultimately, SAARC countries must demonstrate explicit commitment to SOTA care by including it under “Health” in any future SAARC Summit Declarations.

**Table 2:** Implications of SOTA care access across multiple SAARC areas of cooperation (170).

SAARC area of cooperation	SOTA care connections
Human resource development and tourism	Keeping with the region’s needs (see text), SAARC Tourism Ministers should be made aware of the expanding SOTA care tourism to improve access. Building on past bodies, SAARC should introduce dedicated apex and recognized bodies for the umbrella concept of SOTA care. The meeting of SAARC Cabinet Secretaries can include an agenda item on the role of SOTA care in social development.
Agriculture and rural development	Improving SOTA care access for those living in rural and remote regions should be prioritized as a public health issue in this area. The SAARC Development Fund should include scaling up SOTA care infrastructure, including the SAO workforce in rural regions. The quantity and quality of scale-up should be in line with population needs. SOTA care needs of those working in agriculture in rural regions require highlighting. E.g., the burden of specific risk factors and injuries among agricultural workers, financial risk protection in case of care-seeking, etc. Agriculture and Rural Development ministries should be made aware of SOTA care issues that impact their constituents.
Environment, natural disasters, and biotechnology	Delivering SOTA care services have a large environmental impact. SAARC environment and climate change action plans, statements, declarations, conventions, etc. should include assessments of the environmental impact of SOTA care delivery and mitigation and sustainable scale-up strategies. Mass casualties and injuries are unavoidable in natural disasters. Essential SOTA care services should be an integral part of any disaster management or response planning. SOTA care innovations such as low-cost surgical equipment and pain management drugs are integral to medical biotechnology and should be discussed in the Working Group on Biotechnology meetings for inclusion in the National State-of-the-Art Reports on Biotechnology from SAARC.
Economy, trade, and finance	SAARC’s efforts to reduce/remove trade barriers, improve trade facilitation, and enhance investment cooperation should include SOTA care technical innovations and services. SAARC’s focus on enhanced intra-regional investment for improving connectivity by roads, rail, waterways, etc. should include timely geographical access to SOTA care under indirect benefits to strengthen the agenda. Finance ministries should be made aware of the economic and societal benefits of scaling up SOTA care.
Social affairs	Access to SOTA care can improve population health outcomes. Hence, establishing a center for SOTA care, including it in SAARC Health Ministers’ meetings, and integrating it into projects such as the telemedicine network project should be urgently considered (see text for details).
Information and poverty alleviation	SAARC’s initiatives for poverty alleviation must note the high risk for impoverishment among healthcare seekers with a special focus on SOTA care. SAARC’s focus on universalizing telecommunication access should include the spillover impact on tele- and mobile-health interventions that can benefit SOTA care to strengthen the agenda.
Energy, transport, science, and technology	SAARC Transport Ministers should be made aware of the SOTA care geographical inaccessibility issues to ensure inclusion in the high-level meetings. SOTA care innovations should be highlighted in initiatives for industrial research and development and included on a priority basis in mechanisms for intellectual property rights. To reduce inequities in access, SAARC visa liberalization schemes should consider including SOTA care providers.

## 7 Conclusion

South Asia’s path toward universal health coverage necessitates prioritization of and investments in SOTA care. Currently, South Asian countries face several shared and country-specific challenges with limited subnational data on SOTA care indicators and the need for national planning. Case studies of exemplary SOTA care delivery systems, research initiatives, and policy processes are vital for shared learning of countries to be able to tailor the approach toward SOTA care planning. Sustainable and strategic local partnerships under SAARC are of key importance. Evidence-based policymaking, political will, and patients’ participation would ensure SOTA care for all in the region.

## Author contributions

SZ: Conceptualization, Formal analysis, Project administration, Supervision, Resources, Writing – original draft, Writing – review & editing. SR: Methodology, Formal Analysis, Data Curation, Visualization, Validation, Writing – original draft, Writing – review & editing. IG: Data curation, Formal analysis, Methodology, Project administration, Validation, Writing - original draft, Writing – review & editing. NS: Writing – review & editing. CP: Writing – review & editing. AN: Writing – review & editing. HI: Writing – review & editing. AK: Writing – review & editing. AP: Writing – review & editing. GAF: Writing – review & editing. CRKP: Writing – review & editing. C: Writing – review & editing. DS: Writing – review & editing. DG: Writing – review & editing. GJ: Writing – review & editing. IF: Writing – review & editing. JP: Writing – review & editing. JK: Writing – review & editing. LB: Writing – review & editing. MS: Writing – review & editing. MA: Writing – review & editing. NH: Writing – review & editing. NM: Writing – review & editing. NW: Writing – review & editing. PS: Writing – review & editing. SK: Writing – review & editing. SP: Writing – review & editing. TK: Writing – review & editing. TT: Writing – review & editing. VH: Writing – review & editing. DP: Project administration, Writing – review & editing.

## Glossary

UHC: universal health coverage
SOTA: surgical, obstetric, trauma, and anesthesia
WHO: World Health Organization
DCPN: Disease Control Priorities Network
LCoGS: Lancet Commission on Global Surgery
SAARC: South Asian Association for Regional Cooperation
LMICs: lower-middle-income countries
HICs: high income countries
DALY: disability-adjusted life-years
VLW: value of lost welfare
GDP: gross domestic products
SAO: surgery, anesthesia, and obstetric
OBGYN: obstetrics and gynecology
EmOC: met need of emergency obstetric care
MMR: maternal mortality rate
AOC: adequacy of opioid consumption
NSOAPs: national surgical, obstetric, and anesthesia plans
RTI: road traffic injuries
TSS: task-shifting and sharing
PHC: primary health care
